# Supplementary material for: Learning impairments in Fmr1−/− mice on an audio-visual temporal pattern discrimination task
Source: J Neurodev Disord. 2025 Aug 29;17:52. doi: 10.1186/s11689-025-09638-0 (PMC12395853; doi:10.1186/s11689-025-09638-0)

**Supplemental Material**

**Supplementary Figure 1:** *Fmr1^-/-^* mice show variability in learning capacity, splitting into two groups of those who achieve expert performance (d’>2)(n = 5) and those who fail to do so (n = 3). **A.** Percentage of main trial sessions in which mices’ best 150 d-prime (d’) was below 0.5, representative of null discrimination (on average, 9.28 ± 2.70% for WT mice vs. 28.08 ± 6.37% for Expert *Fmr1*^-/-^ mice; p= 0.0264, Mann-Whitney test) (on average, 9.28 ± 2.70% for WT mice vs on average 57.7± 8.95% for Non-Expert *Fmr1*^-/-^ mice; p= 0.0121, Mann-Whitney test). **B.** Number of sessions taken to reach expert performance (d’>2) for *Fmr1^-/-^* (n = 5) and WT mice (n = 8)(on average, 8.38 ± 1.35 d for WT mice vs. 15.2 ± 2.82 d for *Fmr1*^-/-^ mice; p= 0.0317, Student’s t-test).


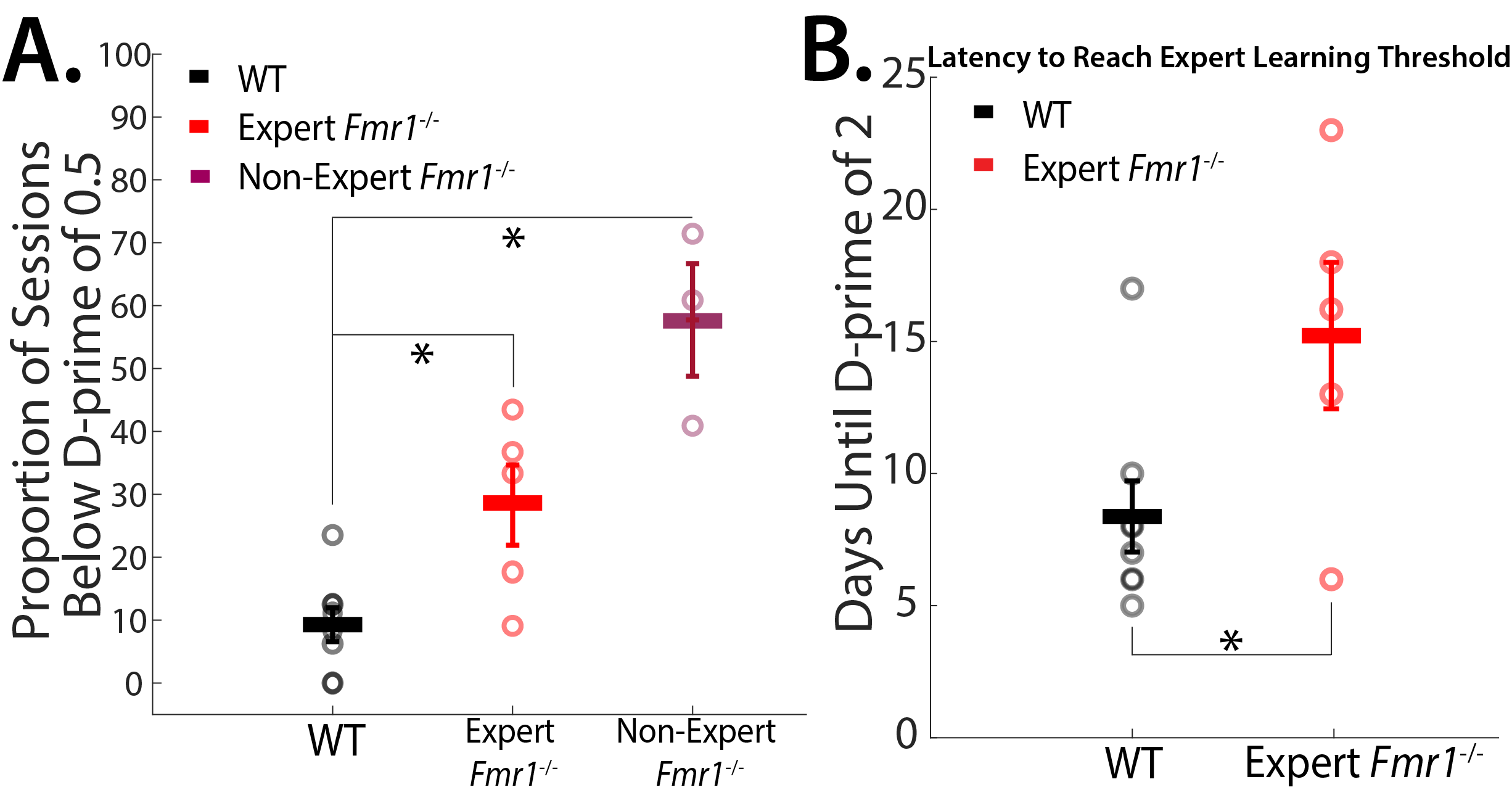


**Supplementary Figure 2:** WT (n = 8) and *Fmr1^-/-^* (n = 8) mice show no differences in pretrial performance. Number of sessions taken to progress past pretrials (on average, 5 ± 0.71 d for WT mice vs. 6.625 ± 1.22 d for *Fmr1*^-/-^ mice; p= 0.2695, Student’s t-test).


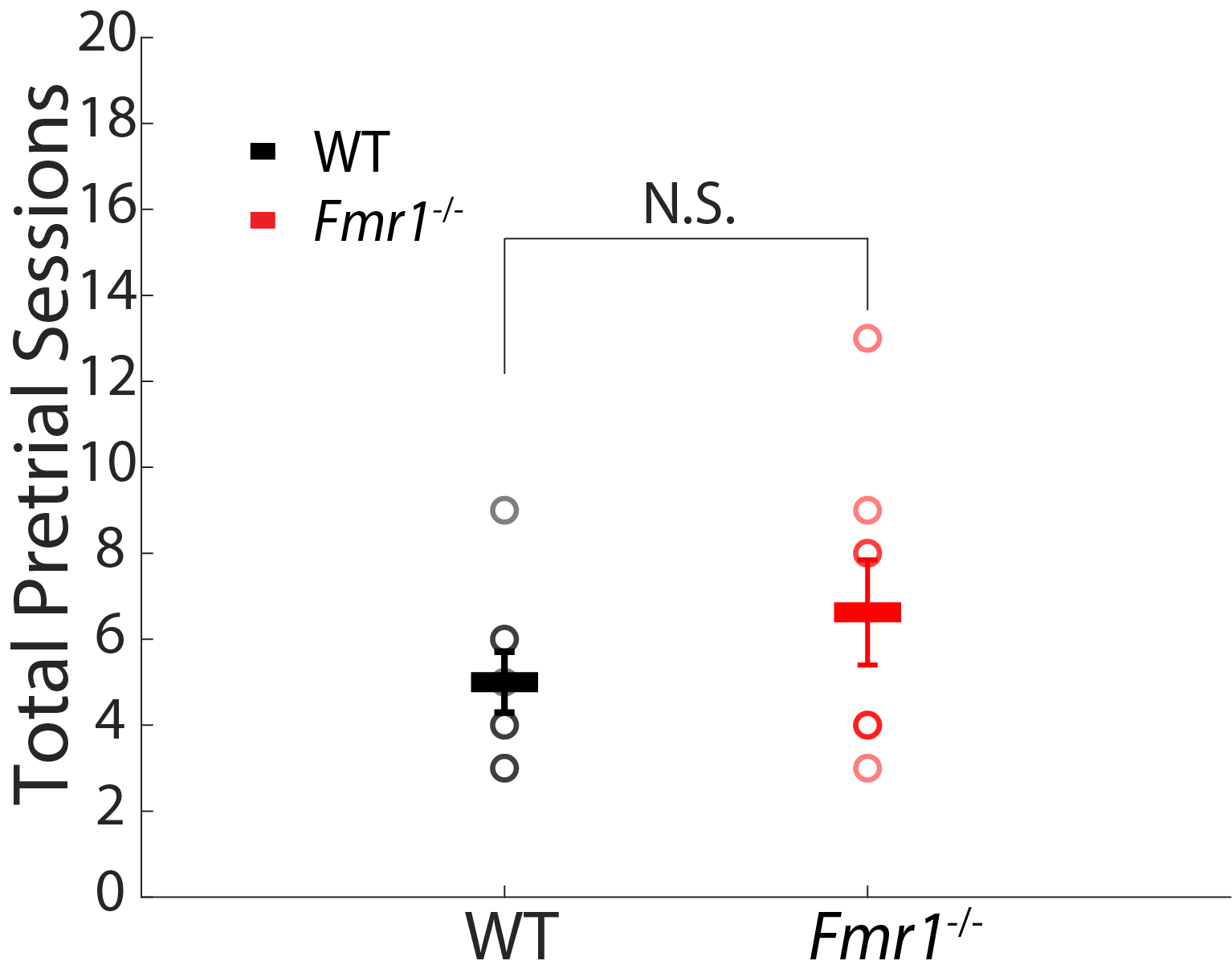


**Supplementary Figure 3:** *Fmr1^-/-^* mice show no difference in learning rate and capacity across sex (n = 3 female, n = 5 male). **A.** Performance across all maintrial sessions broken up by sex.(Mixed-effects analysis for sex effect: p = 0.2849, with Bonferroni correction for multiple comparisons) Performance is measured by the discriminability index (d’). Grey line at d’ = 1 indicates intermediate learning threshold. **B.** Number of sessions taken to reach intermediate performance (d’>1) for Female *Fmr1^-/-^* (n = 3) and Male *Fmr1^-/-^* mice (n = 5)(on average, 12.67 ± 2.19 d for Female *Fmr1^-/-^* mice vs. 9 ± 2.49 d for Male *Fmr1*^-/-^ mice; p= 0.3929, Mann-Whitney Test).


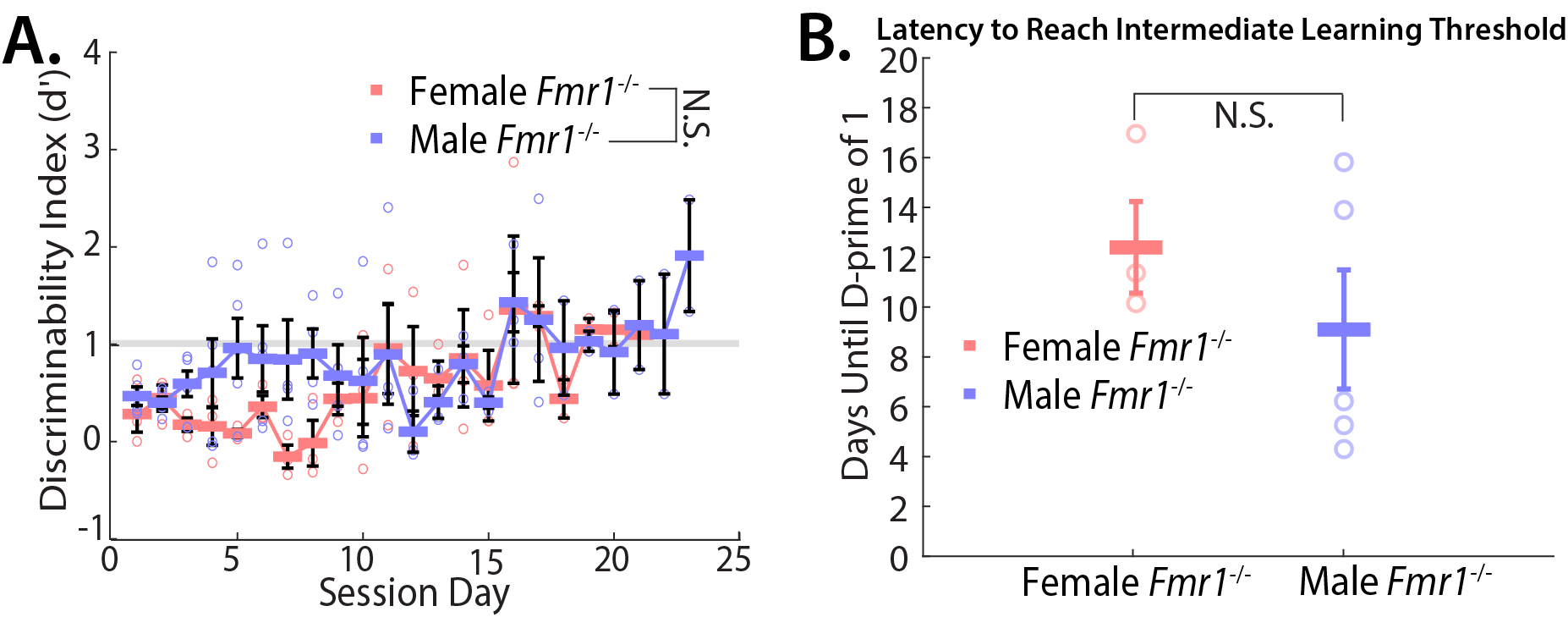

Supplement: Supplementary file 1 — Supplementary Material 1. [file 11689_2025_9638_MOESM1_ESM.docx]
